# An Investigation of the Impact of Precipitation Temperature and Filter Cake Thickness on the Physical Stability of Amorphous Solids: A Case Study

**DOI:** 10.3390/molecules29102327

**Published:** 2024-05-15

**Authors:** Zunhua Li, Xu Liao, Zicheng Gong, Bowen Zhang, Asad Nawaz

**Affiliations:** 1 College of Chemistry and Bioengineering, Hunan University of Science and Engineering, Yongzhou 425199, China; liaoxuhuse@126.com (X.L.); 19574455725@163.com (Z.G.); 007298@yzu.edu.cn (A.N.); 2 School of Chemical Engineering and Technology, Tianjin University, Tianjin 300072, China; bowen_667@tju.edu.cn

**Keywords:** amorphous solid, physical stability, pair distribution function, reduced crystallization temperature, principal component analysis

## Abstract

The purpose of this study was to resolve the issue of physical instability in amorphous solid drugs, which can result in unwanted crystallization, affecting solubility and dissolution rates. The focus was on precipitating physically stable amorphous forms of the nilotinib free base, an anticancer drug, by monitoring preparation conditions such as precipitation temperature and filter cake thickness. A comprehensive set of characterization techniques, including powder X-ray diffraction (PXRD), differential scanning calorimetry (DSC), thermogravimetric analysis (TGA), and focused beam reflectance measurement (FBRM), were used. These were supplemented by advanced data analysis methods that incorporated pair distribution function (PDF), reduced crystallization temperature (*R_c_*), and principal component analysis (PCA) to evaluate the physical stability of the amorphous samples. Results emphasized that optimal physical stability was achieved when amorphous solids were prepared at a precipitation temperature of 10 °C and a filter cake thickness of 4 cm. Moreover, the integration of PDF analysis with *R_c_* values was confirmed as an innovative approach for assessing physical stability, thus offering enhanced efficiency and accuracy over conventional accelerated stability testing methods.

## 1. Introduction

In the pharmaceutical industry, the solid-state form of a drug is crucial for its solubility, dissolution, bioavailability, stability, and therapeutic effectiveness [1,2]. Among the various solid states that drugs can possess, the amorphous state is particularly significant due to its superior properties [3,4]. Drugs in the amorphous state are defined by the absence of long-range order, which sets them apart from their crystalline counterparts. This lack of order endows amorphous substances with greater internal energy, which influences their solubility and dissolution profiles [5,6].

It was essential to recognize that the higher free energy associated with the amorphous state can render drugs more susceptible to physical instability, potentially leading to recrystallization over time [7]. The physical stability of amorphous drugs was influenced by numerous factors. For instance, moisture can function as a plasticizer in amorphous substances, which enhances molecular mobility, thereby promoting recrystallization [8]. This plasticizing effect typically results in a reduction in the glass transition temperature (*T_g_*) of the amorphous solid [9]. This is because water can form hydrogen bonds with polar groups in amorphous drugs, which can significantly affect their *T_g_*. Controlling the moisture content in pharmaceutical formulations was crucial for achieving the desired drug release and bioavailability while also ensuring the physical stability of the drug product.

The preparation temperature exerts a significant influence on the physical stability of amorphous materials, affecting them in various ways. For instance, the fast crystallization tendency (low physical stability) was observed for the amorphous loratadine, which was obtained at a low quench-cooling temperature [10]. Moreover, the thermal history persists in amorphous materials, impacting their long-term stability. Exposure to excessively high temperatures may induce structural relaxation or rearrangement, compromising their stability over time [11]. In addition, minute impurities or residual solvents can initiate crystallization by acting as nucleation sites or disrupting molecular interactions within the amorphous solid, leading to physical instability [12]. Smaller particles, due to their increased surface area-to-volume ratio, were more susceptible to recrystallization because of greater environmental exposure and higher surface energy [13].

The method of amorphization also plays a crucial role in physical stability. Techniques, such as spray-drying, lyophilization, and milling, can create varying degrees of disorder and introduce stresses that may affect long-term physical stability [14,15,16]. Furthermore, parameters in anti-solvent precipitation methods, including residual solvent amount, drying duration, anti-solvent/solvent ratio, anti-solvent feed rate, agitation speed, and aging time, all influence the physical stability of the resulting amorphous solids [17,18]. Mefenamic acid can be efficiently preserved by encapsulating it within the pores of aerogels utilizing supercritical fluid technology. During the integration of these drugs into the aerogel matrix, there was a potential for alterations in their molecular configuration [19]. Therefore, comprehensive understanding and control of these parameters were essential for maintaining the physical stability and performance of amorphous drugs.

Traditionally, the physical stability of solids was evaluated using powder X-ray diffraction (PXRD). However, PXRD patterns of different amorphous solids can be similar, often exhibiting a singular halo peak, making it challenging to distinguish between them [20]. This similarity has rendered the use of PXRD for assessing the physical stability of amorphous drugs less precise. Consequently, there has been a pressing need to develop new methods for evaluating the physical stability of amorphous drugs. Previous studies have shown that the physical stability of amorphous solids can be effectively assessed by converting PXRD data into pair distribution functions (PDFs) and employing principal component analysis (PCA) to discern differences between the resulting PDF data sets [17,18]. Additionally, the reduced crystallization temperature (*R_c_*) value serves as another indicator of the physical stability of an amorphous solid [17,18,21]. A higher *R_c_* value indicates greater resistance to crystallization and a stronger barrier against the transition from an amorphous to a crystalline state.

In the current study, nilotinib free base, an anticancer agent, was employed as a mode compound. The crystalline form of the nilotinib free base exhibits limited solubility, which impedes its bioavailability [22,23]. To circumvent this limitation, an array of formulation strategies has been investigated to enhance its solubility, encompassing spray-dried amorphous solid dispersions and nanoparticles [24,25,26]. The objective of this study was to precipitate amorphous solids of nilotinib free base by manipulating the precipitation temperature and filter cake thickness. The physical stability of the resulting amorphous solids was assessed using the PDF data and *R_c_* values. Furthermore, PCA was applied to all amorphous samples to identify the optimal precipitation conditions for achieving amorphous solids with improved physical stability.

## 2. Results and Discussion

### 2.1. Interatomic Distance of Nilotinib Free Base Molecule

**Figure 1:**
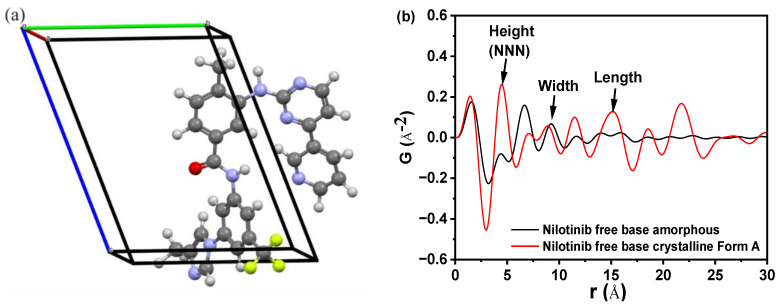
(**a**) Crystal structure of nilotinib free base crystalline Form A (blue = nitrogen, green = fluorine, red = oxygen, white = hydrogen, grey = carbon; a = 4.52 Å, b = 10.64 Å, c = 13.70 Å); (**b**) Comparing the PDF of nilotinib free base crystalline Form A and amorphous form. (The nearest neighbor distances of 4.62, 9.43, and 14.35 Å correspond to the nilotinib free base crystalline Form A’s crystal lattice dimensions (height, width, and length, respectively), representing random close packing governed by molecular shape anisotropy).

Figure 1a demonstrates the crystal structure of nilotinib free base crystalline Form A [27]. This form crystallized in space group *P*1, with lattice parameters *a* = 4.52 Å, *b* = 10.64 Å, *c* = 13.70 Å, *α* = 68.86°, *β* = 82.15°, *γ* = 84.20°, volume *V* = 607.62 Å^3^, and *Z* = 1. The dimensions of the crystal lattice of the nilotinib free base crystalline Form A were determined to be 4.52, 10.64, and 13.70 Å for the height, width, and length, respectively. Figure 1b displays several peaks in the PDF that correspond to the interactions between nearest neighbors. A comparison was made between the crystalline Form A of nilotinib free base and its amorphous solid. The analysis revealed significant alterations in the local nearest neighbor molecular configuration, suggesting a transition from a crystalline to an amorphous packing structure. The arrows indicated that the three nearest neighbor peaks correspond to atom-to-atom distances of 4.62 Å, 9.43 Å, and 14.35 Å. Additionally, the nilotinib free base crystal lattice in the crystalline Form A structure exhibited dimensions of 4.52 Å in height, 10.64 Å in width, and 13.70 Å in length. These measurements closely align with the three nearest neighbor distances determined from the PDF data. The distances observed suggest that the average local structure of the amorphous phase of nilotinib free base was randomly close-packed and influenced by the anisotropy of molecular shape.

Additionally, in the comparison of the PDF data of the amorphous form and crystalline Form A of nilotinib free base, it was observed that crystalline Form A exhibited greater fluctuations than the amorphous form. The intensity of the second peak in the amorphous form (also known as the next nearest neighbor (NNN) peak, which corresponds to the crystal lattice height of the nilotinib free base crystalline Form A) was significantly lower compared to the intensity of the second peak in crystalline Form A. This observation suggested that the degree of disorder in the amorphous form was significantly lower compared to that in crystalline Form A. Similar findings have been reported in prior research conducted on other drugs. For example, it has been deduced that the first and second peaks observed in the PDF can be attributed to the γ crystalline form of indomethacin, representing the molecular height and width, respectively. Additionally, the third peak in the PDF corresponds to the γ crystalline form of indomethacin, specifically indicating the molecular length of the dimer [28,29]. In the PDF of piroxicam, the distances of 12.3 Å, 7.9 Å, and 4.9 Å correspond to the crystal lattice dimensions (height, width, and length, respectively) of piroxicam in its crystalline form [30].

### 2.2. Calibration of Measurement Techniques

**Figure 2:**
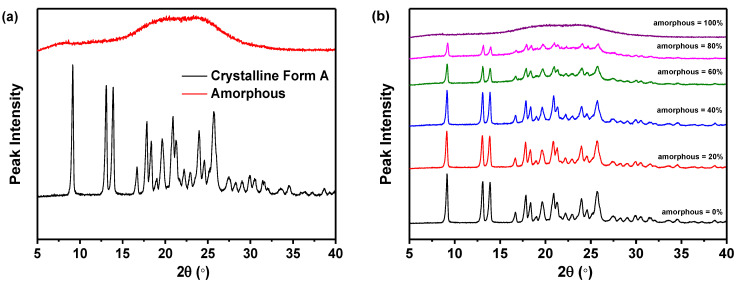

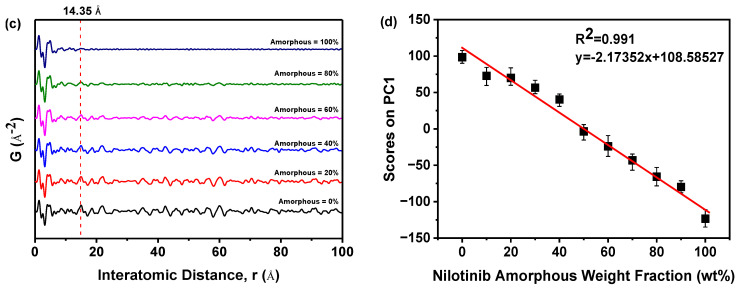
Calibration of measurement techniques: (**a**) PXRD patterns of the nilotinib free base pure crystalline Form A and pure amorphous form; (**b**) PXRD patterns of the mixture samples; (**c**) PDF traces (14.35 Å is corresponding to the length of the crystal lattice dimension for nilotinib free base crystalline Form A); (**d**) Calibration of nilotinib free base amorphous weight fraction with PCA scores on PC1 (*n* = 3).

As presented in Figure 2a, the PXRD patterns of the pure crystalline Form A of nilotinib free base exhibited distinct peaks at 9.07°, 13.08°, 13.82°, 16.68°, 17.85°, 18.26°, 20.84°, 21.39°, 24.11°, and 25.22°. This observation was in complete accordance with the characteristic peaks exhibited by nilotinib free base crystalline Form A, as documented in prior research studies [31,32]. However, the amorphous form exhibited only a halo peak. As depicted in Figure 2b, the PXRD patterns demonstrated that the characteristic peak height of crystalline Form A gradually diminished and eventually disappeared entirely with the progressive increase in the weight fraction of the amorphous form of nilotinib free base in the mixed samples. This observation suggested a corresponding increase in intermolecular disorder.

As revealed in Figure 2c, the PDF of each sample exhibited no significant variation in the interatomic distance when it was below 14.35 Å. The PDF of each sample exhibited a notable disparity in the interatomic distance, specifically above 14.35 Å. As the proportion of amorphous form in the sample increased incrementally, the fluctuation in the PDF decreased. This finding indicated that PDFs with interatomic distances less than 14.35 Å encompassed both intramolecular and intermolecular structural details of the nilotinib free base. Consistent with the longest intramolecular interatomic distance (14.35 Å) of nilotinib free base, as shown in Figure 1b. However, the PDF data with a distance greater than 14.35 Å exclusively encompassed intermolecular structural information. At the same time, this also implied that the disorder in the sample gradually increased as the weight fraction of amorphous material increased.

In addition, the PDF interatomic distance range of 0–15 Å was utilized to calibrate the PCA. The study revealed a negative correlation between the amorphous weight fraction of nilotinib free base and the PCA scores on PC1. As the amorphous weight fraction increased, the PCA scores on PC1 decreased. The correlation between the amorphous weight fraction in the mixed samples and the PCA scores on PC1 was found to be significant (R^2^ = 0.991), indicating a strong linear relationship (Figure 2d). This phenomenon illustrated that by employing optimized conditions for assessing the degree of disorder and physical stability of an amorphous nilotinib free base, more reliable data can be obtained for PXRD, PDF, and PCA. These optimized conditions included a PXRD diffraction angle range of 5–40°, the use of a semiconductor PXRD detector, and a PDF interatomic distance range of 0–15 Å. Thus, a more precise and expedited evaluation of the degree of disorder and physical stability of an amorphous nilotinib free base can be achieved.

### 2.3. Effect of Precipitation Temperature

#### 2.3.1. PDF and PCA of PXRD Data

**Figure 3:**
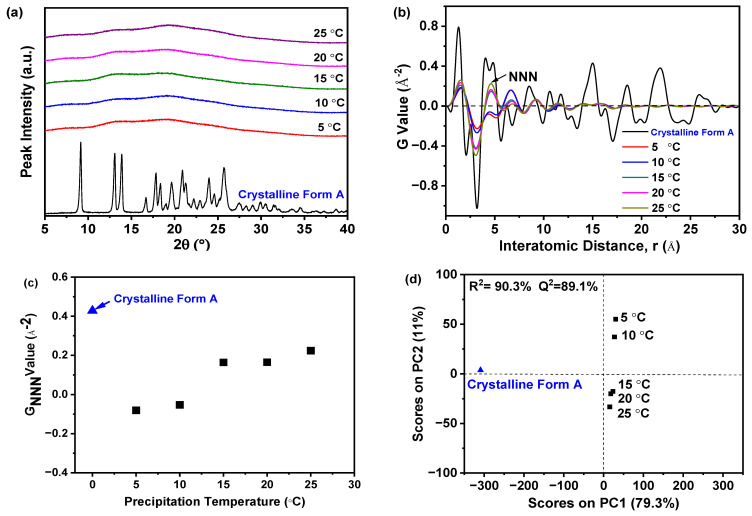
The effect of precipitation temperature on the precipitation nilotinib free base solid samples: (**a**) PXRD patterns; (**b**) PDF traces (The NNN represents the next nearest neighbor atoms in the nilotinib free base solid samples); (**c**) G_NNN_ values; (**d**) PCA scores plot (The R^2^ represents the goodness of fit, while the Q^2^ represents the goodness of prediction). (The crystalline Form A was used as a reference sample, and the filter cake thickness was fixed at 4 cm).

Figure 3a illustrated that samples precipitated at various temperatures exhibited a solitary halo peak, which was characteristic of the amorphous state of a nilotinib free base. Conversely, the PXRD patterns of the reference sample crystalline Form A exhibit distinct peaks at 9.07°, 13.08°, 13.82°, 16.68°, 17.85°, 18.26°, 20.84°, 21.39°, 24.11°, and 25.22° [31,32]. While PXRD effectively differentiates crystalline from amorphous forms, it was challenging to discern between different amorphous samples using PXRD patterns. Therefore, to detect subtle differences among amorphous solids, PXRD data were converted using PDF analysis, as presented in Figure 3b. The distinctive peak at an atomic distance of 4.62 Å, corresponding to the NNN peak in the nilotinib free base PDF trace, was utilized to assess the degree of the disorder. It is worth mentioning that the distance of 4.62 Å corresponds to the molecular height of nilotinib free base in its crystalline Form A [26]. In the research on indomethacin, it was also confirmed that the NNN peak corresponds to the molecular coordination sphere [33]. The variations observed in the PDF traces for samples precipitated between 5 and 25 °C indicate that higher precipitation temperatures result in increased peak heights, with the maximum height at 25 °C. This suggests that the most ordered amorphous solid was obtained at the highest temperature [29,33]. This phenomenon occurs because higher temperatures enhance molecular mobility, promoting a propensity toward crystallization. Consequently, precise temperature control during precipitation was crucial to maintain the amorphous state [34]. Moreover, below the *T_g_*, molecular mobility and the subsequent molecular rearrangements that could lead to nucleation and crystal growth were minimized [35].

The degree of disorder within the samples can be accurately measured using the G_NNN_ value, as depicted in Figure 3c. Samples that precipitated at temperatures ranging from 15 to 25 °C exhibited higher G_NNN_ values compared to those precipitated between 5 and 10 °C. Additionally, a slight fluctuation in G_NNN_ values was noted for the two samples precipitated at 5 and 10 °C. The data indicated that lower precipitation temperatures resulted in a greater degree of disorder when compared to higher precipitation temperatures. Furthermore, to effectively evaluate the differences among these samples, PCA was performed using the previously mentioned PDF data, as shown in Figure 3d. Generally, two principal components (PCs) were sufficient to explain 90.3% of the variance in the PDF data. The first PC (PC1) was responsible for over 79% of the total variance. The second PC (PC2) accounted for 11% of the variation. Although this percentage was relatively small, PC2 seemed to be the crucial component that differentiated whether the reference sample (crystalline Form A) and the prepared amorphous solids had ‘matching’ or ‘non-matching’ PDF profiles in this analysis. Upon visual examination, the loading plots for both PCs derived from these amorphous solids exhibited wave-like patterns (results not shown), resembling the PDF profiles. Furthermore, comparisons between samples from different precipitation temperatures and the reference sample (crystalline Form A) allowed for their classification into three groups: the first included the reference sample, the second comprised samples from 5 and 10 °C precipitation temperatures, and the third consisted of samples from temperatures above 15 °C. Notably, samples precipitated between 15 and 25 °C were distinctly separated from those at lower temperatures in the PCA plot, with the 25 °C sample closest to the reference sample on PC1. This suggests that, of all samples, the one prepared at 25 °C most closely resembled the crystalline Form A in terms of nilotinib free base molecular disorder, aligning with previous findings [17,18]. Moreover, the results revealed a high correlation (R^2^ = 90.3%), indicating a strong concordance between observed and predicted values. The predictive accuracy (Q^2^) reached 89.1%, confirming the high precision in forecasting future outcomes.

Previous research has shown that the impact of precipitation temperature on the physical stability of amorphous solids was primarily determined by the increased molecular motion at elevated temperatures. This can diminish intermolecular forces and potentially compromise physical stability. For instance, enhanced molecular motion may trigger recrystallization in amorphous drugs [36]. Therefore, the experimental findings suggested that reducing the precipitation temperature to 10 °C favored the precipitation of a more physically stable amorphous solid in this study.

#### 2.3.2. *R_c_* Analysis of DSC Data

**Figure 4:**
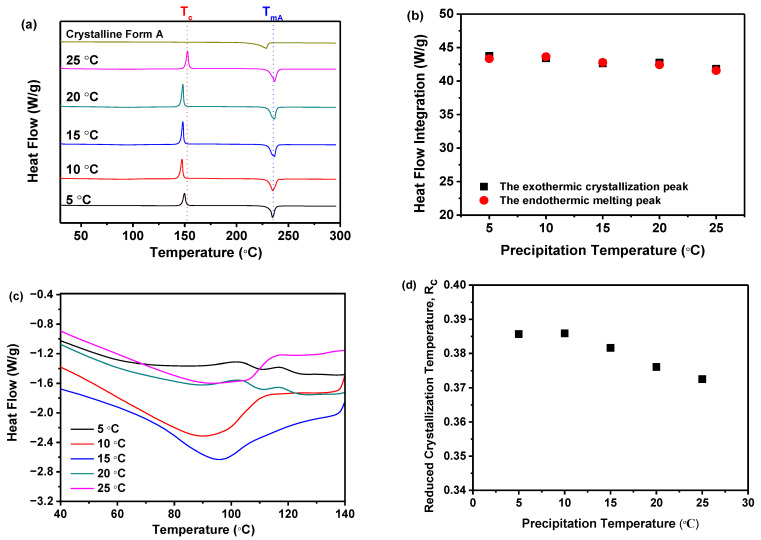
The effect of precipitation temperature on the precipitation nilotinib free base solid samples: (**a**) DSC curves (The *T_c_* represents the precipitation temperature of the amorphous solid samples, while the *T_mA_* represents the melting temperature of nilotinib free base crystalline Form A); (**b**) Comparison of heat flow integration between exothermic crystallization peak and endothermic melting peak; (**c**) Glass transition temperature (*T_g_*); (**d**) Reduced crystallization temperature (*R_c_*). (The crystalline Form A was used as a reference sample, and the filter cake thickness was fixed at 4 cm).

DSC measurement was performed to corroborate the findings from the PXRD analysis. As demonstrated in Figure 4a, all samples, prepared at varying precipitation temperatures, displayed a distinct endothermic peak indicative of the melting point (*T_m_*) for nilotinib free base crystalline Form A (*T_mA_*, 235 °C) [31,32]. Notably, *T_mA_* remained constant across the five samples, irrespective of the differences in precipitation temperature. An exothermic peak around 150 °C was also evident, signifying the transition from the amorphous to the crystalline form, consistent with the previously determined crystallization temperature (*T_c_*) [17,18]. Before the DSC measurements, it was hypothesized that all precipitated samples consisted of amorphous solids. As the temperature increased during the DSC measurements, these amorphous solids underwent a transformation into the crystalline Form A. If a small portion of crystalline Form A was present in the sample before heating, the amorphous solids were more susceptible to crystallization during heating, resulting in a lower *T_c_* compared to other samples. However, as depicted in Figure 4a, no significant variation in *T_c_* was observed among the five samples despite the differences in precipitation temperature used. This comprehensively validated our initial hypothesis, which posited that the samples obtained at varying precipitation temperatures were amorphous and devoid of crystalline structures.

Furthermore, the extrapolated onset temperature for the reference sample (crystalline Form A) was approximately 210 °C, which differed from the other five samples obtained under different precipitation temperatures, with an extrapolated onset temperature of around 218 °C. This indicated that the melting peak of the reference sample (crystalline Form A) was shifted to a lower temperature than that of the other samples. Consequently, it was speculated that the solid that crystallizes from the amorphous phase during heating might be composed of another polymorph. Therefore, the PXRD pattern of the reference sample (crystalline Form A) was compared with that of the other samples heated to a temperature between the exothermic peak and the endothermic peak. It was revealed that no differences were observed (results not shown), which means they were all crystalline Form A. Moreover, by comparing the heat flow integration between the exothermic crystallization peak and the endothermic melting peak, as shown in Figure 4b, it was demonstrated that these two values coincided for the five samples, indicating that these samples were indeed entirely amorphous solids. This finding was also corroborated by PXRD, as shown in Figure 3a.

Moreover, it was important to highlight that the *T_g_* of all samples cannot be identified in Figure 4a. So, the DSC curves within the temperature range of 40 to 140 °C were magnified, as illustrated in Figure 4c. It was observed that there was no discernible pattern of change in the *T_g_* values among these samples. The results indicated that the precipitation temperature did not have a significant impact on the *T_g_* value of these five samples.

Accordingly, as previously mentioned, if the reduced crystallization temperature (*R_c_*) value of a sample was higher than that of other samples, it indicates that this particular sample exhibited superior resistance to transitioning from an amorphous solid to a crystalline form. This means that the sample demonstrates higher physical stability compared to the other samples [21]. Therefore, Figure 4d elucidated that the *R_c_* was influenced by the precipitation temperature. Samples precipitated at 5 and 10 °C exhibited minimal variation in *R_c_*, suggesting that their physical stability was minimally perturbed. However, as the precipitation temperature increased from 15 to 25 °C, the *R_c_* value decreased consistently. This suggests that at higher precipitation temperatures, the physical stability diminishes progressively. Amorphous solids, characterized by elevated interfacial energy, were susceptible to phase separation or aggregation due to temperature-induced modifications in thermodynamic stability [37]. Perturbations in the thermodynamic phase diagram of amorphous solids can impact material physical stability and behavior. Moreover, temperature fluctuations may affect intermolecular forces such as hydrogen bonds and van der Waals interactions, potentially culminating in structural relaxation or rearrangement [11]. For instance, the interactive forces between certain molecules may diminish at elevated temperatures, resulting in structural relaxation or rearrangement of the amorphous substance. Consequently, precipitation temperature dictates amorphous solid physical stability via these mechanisms. Thus, the DSC measurement implies that temperatures below 10 °C favor more physically stable amorphous solids in comparison to those above 15 °C.

#### 2.3.3. Filtering Rate Analysis

**Figure 5:**
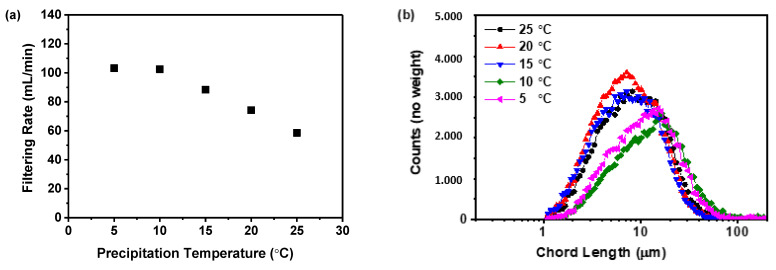

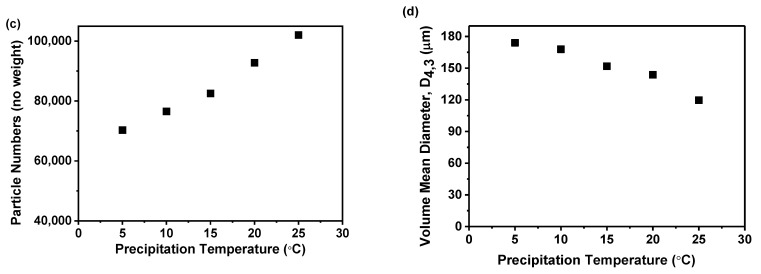
The effect of precipitation temperature on the precipitation nilotinib free base solid samples: (**a**) Filtering rate; (**b**) Particle size distribution; (**c**) Particle numbers; (**d**) Volume mean diameter, D_4,3_. The filter cake thickness was fixed at 4 cm.

The correlation between precipitation temperature and the filtration rate of the precipitated suspension is displayed in Figure 5a. The data indicates a decrease in filtration rate as the precipitation temperature increases. Specifically, raising the temperature from 5 to 25 °C results in a steady decline in the filtration rate from 103.21 to 58.51 mL/min. Moreover, the filtration rate of the samples obtained at precipitation temperatures of 5 and 10 °C was higher than those obtained at precipitation temperatures above 15 °C. This trend was likely due to higher temperatures producing finer solid particles within the suspension, which can slow down the filtration process, a finding consistent with previous research [38]. To confirm this hypothesis, the particle size distribution was measured using focused beam reflectance measurement (FBRM), as shown in Figure 5b,c. The findings indicated that higher precipitation temperatures resulted in smaller particles and an increase in particle number. This relationship between precipitation temperature and particle size was further evidenced by the volume mean diameter calculated from the FBRM data, as displayed in Figure 5d. The analysis showed a direct correlation between precipitation temperature and reduction in particle size. Specifically, rising from 5 to 25 °C led to a decrease in the volume mean diameter from 174.01 to 119.65 μm.

Previous research has indicated that the size and number of particles significantly affect the duration of separation processes [38,39]. Therefore, in the current study, the times of precipitation and storage until filtration were no more than 30 min; a decreased filtration rate could prolong the process and might jeopardize the physical stability of the amorphous solids. This was because an extended filtration period could promote the transformation of an amorphous solid into its crystalline form. During the filtration process, the presence of dimethyl sulfoxide (DMSO), a good solvent for nilotinib free base, in the suspension can lead to the rapid conversion of the prepared amorphous form into its crystalline form if DMSO is not promptly removed by filtration and washing. This finding has been substantiated in our previously published work [18]. Furthermore, both the nucleation and growth stages of the amorphous solid were temperature-dependent. Elevated temperatures can accelerate nucleation, resulting in a greater number of nuclei but with less solute per nucleus, leading to smaller particles. In contrast, lower temperatures decelerate nucleation, producing fewer nuclei with more solute available for growth, culminating in larger particles [40]. Smaller amorphous particles, due to their high surface energy and increased surface area, tend to cluster to minimize free energy. This can lead to aggregation and sedimentation, affecting the physical stability of the amorphous material. The observed phenomenon can be attributed to the incorporation of the good solvent, DMSO, within the aggregating amorphous particles of nilotinib free base during the process of agglomeration. This inclusion facilitates the transition of amorphous solids into their crystalline counterparts. Based on these mechanisms, a precipitation temperature of 10 °C was determined to be optimal for the filtration rate in this study.

Although both 5 and 10 °C were suitable for preparing physically stable amorphous solids, it was considered that maintaining the temperature at 5 °C requires more refrigerant and consumes more energy. Therefore, when considering both physical stability and energy consumption, it was revealed that a precipitation temperature of 10 °C was more appropriate.

### 2.4. Effect of Filter Cake Thickness

#### 2.4.1. PDF and PCA of PXRD Data

**Figure 6:**
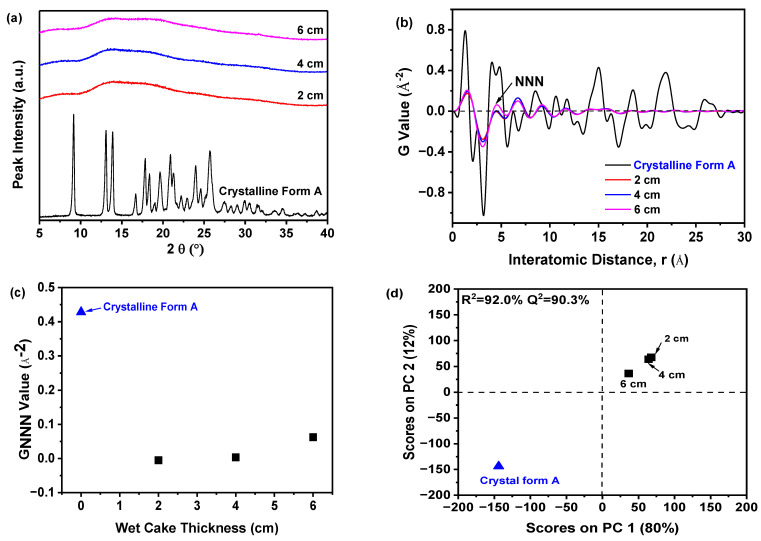
The effect of the filter cake thickness on the precipitation of nilotinib free base solid samples: (**a**) PXRD patterns; (**b**) PDF traces (The NNN represents the next nearest neighbor atoms in the nilotinib free base solid samples); (**c**) G_NNN_ values; (**d**) PCA scores plot (The R^2^ represents the goodness of fit, while the Q^2^ represents the goodness of prediction). (The crystalline Form A was used as a reference sample; the precipitation temperature was fixed at 10 °C).

Figure 6a demonstrated that all samples displayed a consistent halo peak in their PXRD patterns, suggesting an amorphous state, regardless of the filter cake thickness. Due to the difficulty in distinguishing amorphous solids based on PXRD patterns, variations in these amorphous samples could be discerned using PDF analysis of PXRD data, as shown in Figure 6b. The sample prepared with a filter cake thickness of 6 cm exhibited the most significant fluctuations in the PDF curve and had the highest NNN peak compared to samples with 2 cm and 4 cm filter cake thicknesses. As the thickness decreased, the NNN peak height reduced, indicating that thinner thickness may increase the disorder of the amorphous solid samples. Additionally, Figure 6c demonstrated that the degree of disorder was quantified using the G_NNN_ value. The samples with 2 and 4 cm filter cake thicknesses showed lower G_NNN_ values than those with a 6 cm thickness, suggesting they possessed greater disorder. Notably, minimal variation in G_NNN_ values was observed in samples prepared with a thickness of 2 and 4 cm, indicating that 4 cm thickness was sufficient for achieving a higher degree of disorder. Therefore, there was no requirement for a filter cake thicker than 4 cm.

An exhaustive assessment to discern between the amorphous samples was executed utilizing PCA on PDF data, as illustrated in Figure 6d. In general, two PCs were used to achieve a 92% explained variance for the PDF data. The PC1 accounted for 80% of the total variance. The PC2 explained 12% of the variation. The PC1 appears to be the crucial component that differentiates whether the reference sample (crystalline Form A) and the prepared amorphous solids’ PDF profiles matched or did not match in this analysis. Furthermore, the results indicated a strong correlation between observed and predicted values, with an R^2^ of 92.0% and Q^2^ at 90.3%, demonstrating high predictive accuracy. The PCA outcomes revealed exceptional fitting and prediction, enabling categorization into three distinct groups. As shown in Figure 6d, the reference sample (crystalline Form A) constituted the first group; a solitary sample with a 6 cm thickness formed the second group, while the third group encompassed samples with 2 and 4 cm thicknesses, exhibiting pronounced structural disparities from the others. The deviation along PC1 from the reference sample (crystalline Form A) was substantial in the third group, suggesting a distinctive solid structure.

Previous investigations have demonstrated that the effect of filter cake thickness on the physical stability of amorphous solids was primarily mediated by its impact on intergranular forces and the internal pressure distribution within the solids [41]. Modifications in the filter cake thickness can influence the pressure distribution during the filtration process. A thicker cake may result in a larger pressure gradient, potentially leading to structural non-uniformity and compromising the physical stability of the amorphous solid [42]. Consequently, our experimental findings suggested that reducing the filter cake thickness to 4 cm resulted in a more physically stable amorphous solid in this study.

#### 2.4.2. *R_c_* Analysis of DSC Data

**Figure 7:**
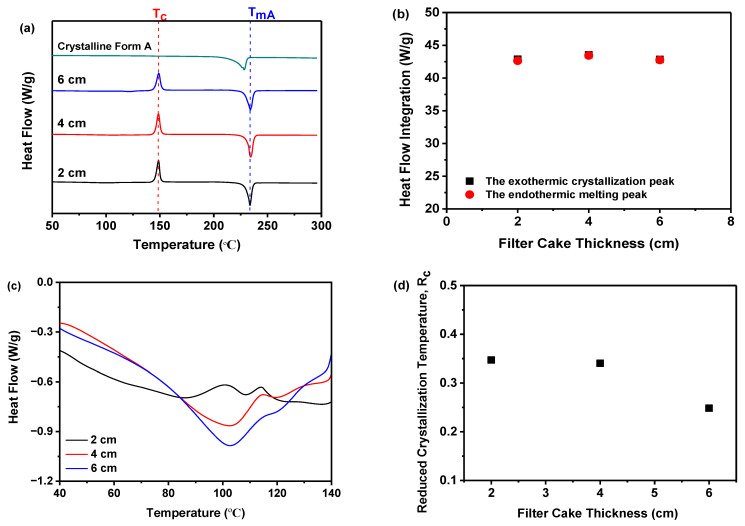
The effect of the filter cake thickness on the precipitation of nilotinib free base solid samples: (**a**) DSC curves (The *T_c_* represents the precipitation temperature of the amorphous solid samples, while the *T_mA_* represents the melting temperature of nilotinib free base crystalline Form A); (**b**) Comparison of heat flow integration between exothermic crystallization peak and endothermic melting peak; (**c**) Glass transition temperature (*T_g_*); (**d**) Reduced crystallization temperature (*R_c_*). (The crystalline Form A was used as a reference sample; the precipitation temperature was fixed at 10 °C).

Figure 7a demonstrated that, irrespective of the filter cake thickness, all samples exhibited a solitary endothermic peak, characteristic of the melting temperature (*T_mA_*, 235 °C) of nilotinib free base crystalline Form A [30,31]. An exothermic peak at 150 °C, suggestive of the transition from the amorphous state to crystalline Form A, was observed across all samples, corroborated by the *T_c_* of crystalline Form A [17,18]. No significant variation in *T_c_* was evident among samples prepared with varying thicknesses. This comprehensively validated that the samples obtained at varying thickness were amorphous and devoid of crystalline structures. Furthermore, by comparing the heat flow integration between the exothermic crystallization peak and the endothermic melting peak, as shown in Figure 7b, it was demonstrated that these two values coincided for the three samples, indicating that these samples were indeed entirely amorphous solids. This finding was also corroborated by PXRD, as shown in Figure 6a.

In addition, the *T_g_* was also depicted in Figure 7c. It was observed that the *T_g_* values of these samples, prepared under different filter cake thicknesses, did not exhibit any discernible pattern of change. The findings of the study suggest that the filter cake thickness did not exert a statistically significant influence on the *T_g_* of the three samples under investigation. Moreover, the fluctuation in the *R_c_* value relative to the filter cake thickness is shown in Figure 7c. Samples derived from a 6 cm thickness showed the lowest *R_c_*, whereas those from 2 and 4 cm thicknesses displayed higher *R_c_* values. The minor *R_c_* variations between these latter two indicate subtle disparities in physical stability [17,18,20]. During the formation of the filter cake, interparticle forces within amorphous particles may vary with increased thickness. For instance, a thicker filter cake could augment interactions such as van der Waals forces, impacting the physical stability of the amorphous solids [43]. Optimizing the filter cake thickness can diminish pressure, thereby enhancing the physical stability of the amorphous solids. Hence, the DSC measurement also substantiated that reducing the filter cake thickness to 4 cm resulted in amorphous solids with superior physical stability.

#### 2.4.3. Thermogravimetric Analysis (TGA)

**Figure 8:**
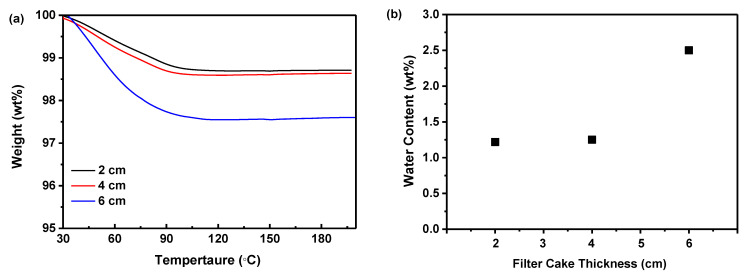
The effect of the filter cake thickness on the precipitation of nilotinib free base solid samples: (**a**) TGA curves; (**b**) Water content. The precipitation temperature was fixed at 10 °C.

Figure 8a illustrated that the weight of the samples progressively decreased as the temperature ascended to 100 °C during TGA measurement, attributed to the evaporation of water contained in the dried samples. However, beyond 100 °C, the absence of significant weight loss suggested the dried samples contained no residual DMSO solvent [44]. An increment in filter cake thickness from 2 to 6 cm correlated with an increase in weight loss, indicating a higher residual water content in thicker samples. Nevertheless, the weight loss was nearly equivalent for 2 and 4 cm thicknesses, demonstrating that a 4 cm thickness was sufficient to minimize the water content. A reduction in thickness below 4 cm did not appreciably decrease the water content in the amorphous solids. Additionally, Figure 8b indicated that increasing the filter cake thickness increased the water content from 1.22 to 2.50%. While decreasing the thickness lowered the water content in the dried samples, there was little discernible difference between 2 and 4 cm, confirming that a 4 cm thickness effectively reduces water content. Since moisture can plasticize amorphous materials, thereby enhancing molecular mobility and crystallization [8], this phenomenon may elucidate why thicker filter cakes result in less physically stable samples.

Although both 2 cm and 4 cm thicknesses were viable for producing physically stable amorphous solids, employing a 2 cm filter cake necessitates increased labor and extended processing time. Consequently, taking into account both physical stability and efficiency, a 4 cm filter cake thickness emerges as the preferable choice.

### 2.5. Accelerated Stability Test

**Figure 9:**
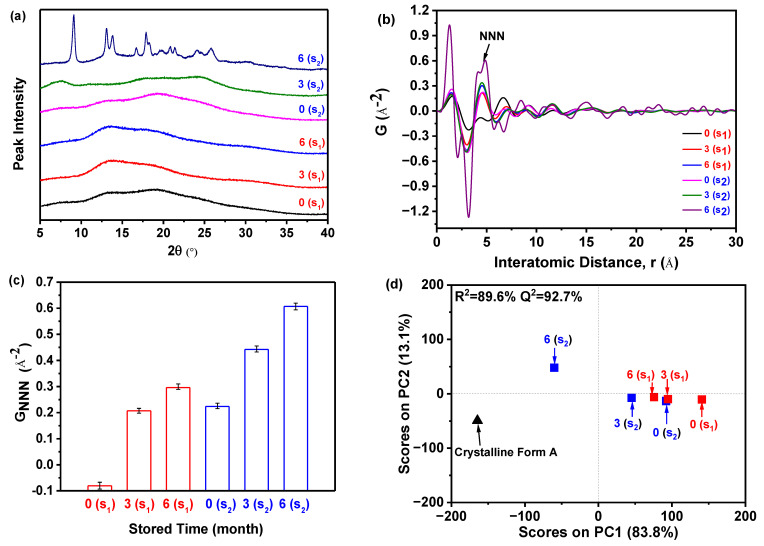
Accelerated stability test: (**a**) PXRD patterns of the samples S_1_ and S_2_ with different storage times in the drug stability test chamber; (**b**) PDF traces (The NNN represents the next nearest neighbor atoms in the nilotinib free base solid samples); (**c**) G_NNN_ values (*n* = 3); (**d**) PCA scores plot (The R^2^ represents the goodness of fit, while the Q^2^ represents the goodness of prediction, the crystalline Form A was used as a reference sample). (0(S_1_), 3(S_1_), and 6(S_1_) represent the sample S_1_ stored for 0, 3, and 6 months, respectively; 0(S_2_), 3(S_2_), and 6(S_2_) represent the sample S_2_ stored for 0, 3, and 6 months, respectively).

As illustrated in Figure 9a, the PXRD patterns demonstrated that sample S_1_, which was prepared at 5 °C and stored for 0, 3, and 6 months, exhibited only a halo peak. This indicated that the sample remained in its amorphous form and did not undergo any crystallization during the accelerated stability test. On the contrary, in the case of sample S_2_ (prepared at 25 °C) stored for 0 and 3 months, the patterns similarly displayed solely a halo peak, suggesting that they maintained their amorphous state. However, after storing sample S_2_ for 6 months, the resulting pattern displayed the distinctive peak associated with the crystalline Form A of nilotinib free base. This peak was observed at angles of 9.07°, 13.08°, 13.82°, 16.68°, 17.85°, 18.26°, 20.84°, 21.39°, 24.11°, and 25.22°. The characteristic peaks observed in this study were found to be in complete agreement with the characteristic peaks previously reported for crystalline Form A of nilotinib free base [30,31]. It was important to highlight that, following a storage period of 6 months, the amorphous sample S_2_ has undergone partial transformation into crystalline Form A. However, a significant portion of the sample remains in its amorphous state. As a result, the PXRD pattern of this sample closely resembled that of the sample shown in Figure 2b. According to the measurement calibration in Figure 2d, it was calculated that the amorphous content was 78%, and the crystalline Form A content was 22% in this sample. This finding indicated that sample S_2_ experienced a phase transformation from its amorphous state to crystalline Form A as the storage time was increased from 3 to 6 months. Accordingly, it was revealed that sample S_1_ exhibited greater physical stability compared to sample S_2_. This conclusion aligns with the outcomes derived from the integration of PDF and *R_c_* in Section 2.3, which thoroughly corroborates the precision and expediency of the methodology employed previously.

However, discerning the distinctions among the amorphous samples solely based on their PXRD patterns proved to be a challenging task. Therefore, the PDF was utilized, as displayed in Figure 9b. The findings of the study revealed that sample S_2_, which was stored for 6 months, exhibited greater fluctuations in PDF and the highest peak height of NNN in comparison to the remaining samples. Additionally, the NNN peak height of the amorphous samples in S_1_ exhibited an increase as the storage time increased from 0 to 6 months. Similarly, the NNN peak height of the amorphous samples in S_2_ showed an increase as the storage time increased from 0 to 3 months.

In addition, the degree of disorder in the samples can also be assessed by utilizing the G_NNN_ value, as illustrated in Figure 9c. For samples S_1_ and S_2_, it was observed that the G_NNN_ value of the sample stored for 6 months was higher compared to those stored for 0 and 3 months. This suggested that a lower G_NNN_ value was indicative of a higher degree of disorder. The sample stored for 0 months exhibited the lowest G_NNN_ value, indicating a higher degree of disorder. Furthermore, upon comparing samples S_1_ and S_2_, both of which were stored for 0 months, it was observed that sample S_1_ exhibited a lower G_NNN_ value. This suggested that sample S_1_ possessed a higher degree of disorder and superior physical stability in comparison to sample S_2_. Hence, it can be observed that sample S_2_ exhibited a higher degree of ease in crystallization compared to sample S_1_ (Figure 9a).

The PCA was conducted using the PDF data to evaluate the degree of disorder, as shown in Figure 9d. In general, two PCs were used to achieve 96.9% explained variance for the PDF data. The PC1 accounted for 83.8% of the total variance. The PC2 explains 13.1% of the variation. The PC1 appears to be the key component that determines whether the reference sample (crystalline Form A) and the prepared amorphous solids’ PDF profiles were ‘matching’ or ‘non-matching’ in this analysis. Moreover, the results indicated a strong correlation between observed and predicted values, with an R^2^ of 89.6% and Q^2^ at 92.7%, demonstrating high predictive accuracy. The PCA outcomes revealed exceptional fitting and prediction, enabling categorization into three distinct groups. As shown in Figure 9d, the samples were categorized into three groups based on the observations made: (1) The crystalline Form A, which served as the reference sample; (2) Sample S_2_, which had been stored for 6 months; (3) The remaining samples. The sample S_2_, which had been stored for 6 months, was found to be positioned separately from the other samples along the PC1 axis and was close to the crystalline Form A. This suggested that it exhibited a higher degree of similarity to the crystalline Form A compared to the other samples. Among the samples belonging to the third category, it was observed that sample S_1_, which had been stored for 0 months, exhibited the greatest deviation from the crystalline Form A. This suggested that it possessed the most distinct solid structure compared to the crystalline Form A and exhibited the highest degree of disorder. Specifically, it can be observed that sample S_1_, which was stored for 0 months, exhibited greater physical stability compared to sample S_2_, which was also stored for 0 months. According to the results of the PCA, it has been confirmed that sample S_1_ exhibited greater physical stability compared to sample S_2_.

In conclusion, the findings of this study suggested that there was no need to wait for 6 months to assess the physical stability of samples through PXRD measurements. Instead, the measurement of the PXRD of the samples during the initial stage of sample preparation, in conjunction with the analysis of the PDF and PCA, can effectively and expeditiously evaluate the degree of disorder and physical stability of amorphous drugs. Hence, the utilization of PXRD, PDF, and PCA techniques offered significant benefits in the precise and efficient evaluation of the degree of disorder and physical stability of amorphous drugs as compared to an accelerated stability test.

### 2.6. Discussion on Future Work

The amorphous form of solid drugs is susceptible to physical instability, which can lead to crystallization and a concomitant reduction in solubility and dissolution rate. Consequently, numerous studies, including the present one, have sought to develop physically stable amorphous formulations of the anticancer agent nilotinib free base by manipulating various parameters pertinent to amorphous precipitation. These parameters included the feeding rate of the anti-solvent, agitation speed, aging time, washing water volume, drying time, anti-solvent/solvent ratio, precipitation temperature, and filter cake thickness [17,18]. The results from these investigations indicated that, under specific conditions, the amorphous solid samples displayed enhanced physical stability. Specifically, optimal physical stability was achieved with a feeding rate of 5 mL/min, an agitation speed of 500 rpm, an aging time of 10 min, a washing water volume of more than 50 mL, a drying time extended beyond 18 h, an anti-solvent/solvent ratio surpassed 40, a precipitation temperature around 10 °C, and filter cake thickness of 4 cm. Furthermore, the innovative approach of employing PXRD data for PCA and calculating the *R_c_* value from the DSC data enabled the assessment of the physical stability of amorphous solid drugs. This method offers inherent advantages in terms of enhanced speed and precision over conventional accelerated stability testing for evaluating the physical stability of amorphous solid samples. Additionally, our research group conducted comparable studies on other biopharmaceutics classification system (BCS) Class II and IV drugs, such as indomethacin, piroxicam, and carbamazepine. The findings suggested that diverse drug molecules may require similar precipitation conditions to attain optimal physical stability. This demonstrated that the optimal preparation conditions for amorphous substances were potentially universal across different drug molecules, and these research findings are forthcoming in subsequent publications.

Furthermore, the amorphous solids of nilotinib free base can be prepared by neutralization with hydrochloric acid. Therefore, it is necessary to examine the influence of hydrochloric acid concentration, pH value, nilotinib free base concentration, and other parameters on the physical stability of the prepared amorphous solids. The study of these parameters will be the focus of our future research. Additionally, it is important to acknowledge that relying solely on physical stability assessment is insufficient for evaluating formulation stability. Chemical stability must also be taken into consideration. The objective of our previous study was to enhance the conditions for the production of physically stable amorphous solids. Subsequent research will focus on examining the chemical and physical stability of nilotinib free base or other drug solids (e.g., indomethacin, piroxicam, and carbamazepine) while further optimizing the preparation conditions for amorphous forms.

## 3. Materials and Methods

### 3.1. Materials

**Figure 10:**
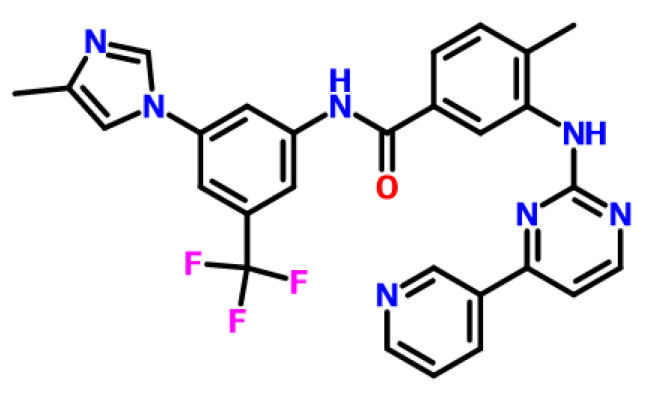
The molecular structure of nilotinib free base, C_28_H_22_F_3_N_7_O.

Heryi Pharma provided a nilotinib free base (purity of ≥98%, Anhui Heryi Pharmaceutical Co., Ltd., Tianchang, China), which was a solid in crystalline Form A, and the molecular structure of nilotinib free base was shown in Figure 10. DMSO was purchased from Titan (purity ≥ 99%, Shanghai Titan Technology Co., Ltd., Shanghai, China). Deionized water was obtained with a Millipore ultrapure water system (Applied Membranes Inc., Vista, CA, USA).

### 3.2. Methods

#### 3.2.1. Precipitation of Nilotinib Free Base Amorphous Solids

**Figure 11:**
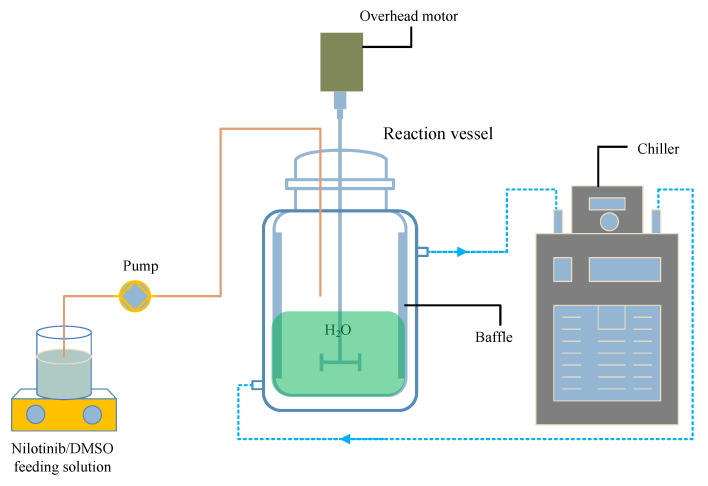
Experimental setup for the preparation of nilotinib free base amorphous solids.

Following our previous studies, as shown in Figure 11, a measured amount of solid nilotinib free base was completely dissolved into DMSO (the solvent) at 70 °C, resulting in a 10 mL solution of nilotinib free base with a concentration of 0.27 mol/L [17,18]. The solution was then filtered to eliminate any residual solid impurities. Afterward, an appropriate volume of deionized water (the anti-solvent) was added to a reaction vessel equipped with four baffles (S300, Beijing Century Senlang Experimental Instrument Co., Ltd., Beijing, China). The deionized water was cooled to temperatures of 5, 10, 15, 20, and 25 °C, respectively, using a chiller (KGDH-2030, Nanjing Kenfan Electronic Technology Co., Ltd., Nanjing, China). With the stirring speed maintained at 500 rpm, the nilotinib free base solution was gradually added to the deionized water in the reaction vessel via a peristaltic pump (BT100FC, Baoding Rongbai Constant Flow Pump Manufacturing Co., Ltd., Baoding, China) at a constant flow rate of 5.0 mL/min to initiate the anti-solvent precipitation process. The ratio of the anti-solvent to solvent volumes was kept constant at 40.

The aqueous solubility of the nilotinib free base was notably low, leading to the rapid precipitation of a solid suspension containing amorphous particles upon mixing the nilotinib free base solution with deionized water. This suspension was kept under continuous stirring and allowed to age for 10 min following the completion of the feed process. Isolation of the wet filter cake of nilotinib free base was achieved using a circulating water vacuum pump (SHZ-III A, Gongyi Ruide Instrument Equipment Co., Ltd., Gongyi, China) in conjunction with qualitative filter paper. During filtration, the wet filter cake was washed with 50 mL of deionized water to eliminate residual DMSO. The washed filter cake was then dried in a vacuum oven (DHG-9055A, Wujiang Yonglian Machinery Equipment Factory, Wujiang, China) at 40 °C for 18 h, resulting in nilotinib free base samples with varying water content. These samples were subsequently placed into capped glass bottles and stored at room temperature until measurement, with each experiment being replicated three times.

#### 3.2.2. Filter Test

The sample suspension was filtered using a vacuum pump (Model SHZ-III A, Gongyi Ruide Instrument Equipment Co., Ltd., Gongyi, China) with varying filter cake thicknesses of 2, 4, and 6 cm. The time elapsed from the start of filtration until no more filtrate passed through the filter paper was documented as the filtration time. The filtration rate was subsequently calculated by dividing the volume of the suspension by the time taken for the filtration process. Each experiment was also conducted three times to ensure reproducibility.

#### 3.2.3. Calibration Experiment

Eleven mixtures were prepared, these samples were physically mixed based on the weight fractions of the respective phases, consisting of a combination of nilotinib free base amorphous form solid and crystalline Form A solid. The weight fraction of the amorphous form solid in these mixtures ranged from 0% to 100%. PXRD measurement was conducted on each mixture using a Bruker D8 powder X-ray diffractometer equipped with a semiconductor detector (Bruker, Karlsruhe, Germany). The PXRD diffraction angle range of 5–40° was employed for the measurement. The PXRD data obtained from the experiment were subsequently subjected to Fourier transformation to generate the PDF data. Finally, the PDF interatomic distance range of 0–15 Å was utilized to conduct PCA. The PCA data were utilized for the calibration of the amorphous solid mass percentage in the mixture. Each experiment was conducted in triplicate.

#### 3.2.4. Characterization of Nilotinib Free Base Solid Samples

##### Powder X-ray Diffraction (PXRD)

The PXRD measurement was promptly conducted on the dried nilotinib free base samples at room temperature using a Bruker D8 instrument equipped with semiconductor detectors (Bruker, Karlsruhe, Germany). The PXRD parameters for this study were as follows: Cu Kα radiation with a wavelength of 1.54 Å, a tube voltage of 100 kV, and a tube current of 80 mA. A consistent step size of 0.02° and a scanning speed of 6°/min was maintained, with scans covering a diffraction angle range from 5 to 40°. Each sample was measured three times.

##### Differential Scanning Calorimetry (DSC)

The thermal properties of the dried samples were analyzed using a DSC instrument (Q2000, TA Instruments, New Castle, DE, USA). Each experiment was repeated thrice for consistency. The temperature ranged from 30 to 300 °C with a heating rate of 10 °C/min. Approximately 5 mg of each sample was sealed in a T161116 disk from TA Instruments and purged with nitrogen at a flow rate of 50 mL/min during heating. From the DSC data, glass transition temperature (*T_g_*), crystallization temperature (*T_c_*), and melting temperature (*T_m_*) were determined. The *R_c_* value was subsequently calculated using the formula as shown below [45]. The *R_c_* value indicates how much above *T_g_* a sample should be heated for crystallization and assesses the crystallization tendency of amorphous solids.

$$R_{c}=\frac{T_{c}-T_{g}}{T_{m}-T_{g}}$$

##### Thermogravimetric Analysis (TGA)

Water content and residual DMSO solvent in the dried samples were determined using TGA performed on a TGA8000 analyzer (PerkinElmer, Waltham, MA, USA). Approximately 5 mg of each sample was placed in an aluminum pan with an open lid. The temperature ranged from 30 to 200 °C at a heating rate of 10 °C/min, while nitrogen was purged at a flow rate of 20 mL/min. Each test was repeated three times for accuracy.

##### Focused Beam Reflectance Measurement (FBRM)

The FBRM instrument (FBRM G400, Mettler Toledo, Columbus, OH, USA) was utilized for continuous monitoring of the particle size of nilotinib free base precipitates. The FBRM probe was directly inserted into the reaction vessel, capturing particle size data every 10 s throughout the feeding process. To ensure consistency, each experiment was repeated three times.

#### 3.2.5. Pair Distribution Function (PDF)

The analysis of the PDF was performed using Fourier transformation on PXRD data, facilitated by the PDFgetX3 software (Version 2.2.1, Columbia University, New York, NY, USA). The configuration details were provided in references [46]. The program can be accessed via https://www.diffpy.org/products/pdfgetx.html (accessed on 26 March 2024). To ensure accuracy, each analysis was repeated three times.

#### 3.2.6. Principal Components Analysis (PCA)

This research employed PCA as a statistical method to discern the disparities in the PXRD and PDF outcomes across diverse amorphous solids. The data preprocessing and scaling were executed using SIMCA 15 software (Version 15.0, Sartorius, Göttingen, Germany), adhering to the methodology detailed by Karmwar et al. [47]. The PCA was limited to interatomic distances spanning from 0 to 15 Å, encompassing the primary peaks indicative of amorphous solids, as previously reported in the literature [48,49], PCA datasets were meticulously collected for each sample, with each analysis being replicated three times to ensure precision.

#### 3.2.7. Accelerated Stability Test

Two distinct amorphous samples of nilotinib free base, which were prepared at the precipitation temperature of 5 and 25 °C, were chosen for the accelerated stability test. The sample prepared at the precipitation temperature of 5 °C was labeled as S_1_, while the sample prepared at the precipitation temperature of 25 °C was labeled as S_2_. Additionally, following the drying process of the amorphous samples of nilotinib free base, each sample was divided into three equal parts. One part of the samples was promptly subjected to PXRD measurement (referred to as 0(S_1_) and 0(S_2_), representing a storage duration of 0 months). Furthermore, the remaining four portions of the samples were promptly transferred into glass bottles without caps and subsequently stored in a drug stability test chamber (YP-250GSP, Shanghai Suying Test Instrument Co., LTD, Shanghai, China) for a duration of 3 and 6 months. These samples were designated as 3(S_1_), 6(S_1_), 3(S_2_), and 6(S_2_), respectively. The temperature and relative humidity within the chamber were maintained at 40 °C and 75%, respectively. Following the prescribed procedure outlined in Section 3.2.4, Section 3.2.5 and Section 3.2.6, the PXRD, PDF, and PCA data of these samples were acquired after the storage periods. Each experiment was conducted in triplicate.

## 4. Conclusions

The primary challenge in formulating amorphous solid drugs was their inherent propensity for physical instability, which leads to undesirable crystallization and concomitant reductions in solubility and dissolution rates. In response to this issue, the present study was meticulously designed for engineering a physically stable amorphous formulation of nilotinib free base by adjusting key precipitation parameters, notably the precipitation temperature and filter cake thickness. The results were compelling, revealing that an optimal blend of a precipitation temperature of 10 °C and a 4 cm filter cake thickness resulted in amorphous samples with enhanced physical stability. Moreover, the study’s methodological innovation, characterized by the application of PDF and PCA on PXRD data, complemented by *R_c_* values derived from the DSC data, proved to be a game-changer in assessing physical stability. This novel approach not only expedited the evaluation process but also delivered more precise and accurate results, outperforming conventional accelerated stability testing methods. This methodological breakthrough could redefine how we assess and ensure the physical stability of amorphous solid drugs, offering pharmaceutical scientists a more efficient and reliable tool to optimize drug formulations. In conclusion, this study has successfully demonstrated that carefully controlling precipitation parameters can produce an amorphous nilotinib free base with improved physical stability. Additionally, adopting advanced analytical techniques, such as PDF, PCA, and *R_c_* values, has great potential to enhance the speed and accuracy of physical stability assessments. These advancements pave the way for more robust and efficacious amorphous drug formulations, ensuring better therapeutic outcomes for patients.

## Data Availability

Data are contained within the article.
